# The Dynamic Intestinal Absorption Model (Diamod®), an *in vitro* tool to study the interconnected kinetics of gastrointestinal solubility, supersaturation, precipitation, and intestinal permeation processes of oral drugs

**DOI:** 10.1016/j.ijpx.2023.100177

**Published:** 2023-03-07

**Authors:** Frédéric Moens, Gies Vandevijver, Anke De Blaiser, Adam Larsson, Fabio Spreafico, Patrick Augustijns, Massimo Marzorati

**Affiliations:** a ProDigest BV, Technologiepark-Zwijnaarde 73, 9052 Ghent, Belgium; b Drug Delivery and Disposition, KU Leuven, Gasthuisberg O&N II, Herestraat 49 - box 921, Leuven 3000, Belgium; c Center of Microbial Ecology and Technology (CMET), Faculty of Bioscience Engineering, Ghent University, Coupure Links 653, 9000 Ghent, Belgium

**Keywords:** Diamod®, Gastrointestinal dissolution, Intestinal permeation, Solubility-permeability interplay, Itraconazole, Indinavir sulfate

## Abstract

This study aimed at developing the Diamod® as a dynamic gastrointestinal transfer model with physically interconnected permeation. The Diamod® was validated by studying the impact of the intraluminal dilution of a cyclodextrin-based itraconazole solution and the negative food effect for indinavir sulfate for which clinical data are available demonstrating that the systemic exposure was strongly mediated by interconnected solubility, precipitation, and permeation processes. The Diamod® accurately simulated the impact of water intake on the gastrointestinal behavior of a Sporanox® solution. Water intake significantly decreased the duodenal solute concentrations of itraconazole as compared to no intake of water. Despite this duodenal behavior the amount of permeated itraconazole was not affected by water intake as observed *in vivo*. Next to this, the Diamod® accurately simulated the negative food effect for indinavir sulfate. Different fasted and fed state experiments demonstrated that this negative food effect was mediated by an increased stomach pH, entrapment of indinavir in colloidal structures and the slower gastric emptying of indinavir under fed state conditions. Therefore, it can be concluded that the Diamod® is a useful *in vitro* model to mechanistically study the gastrointestinal performance of drugs.

## Graphical abstract

**Figure:**
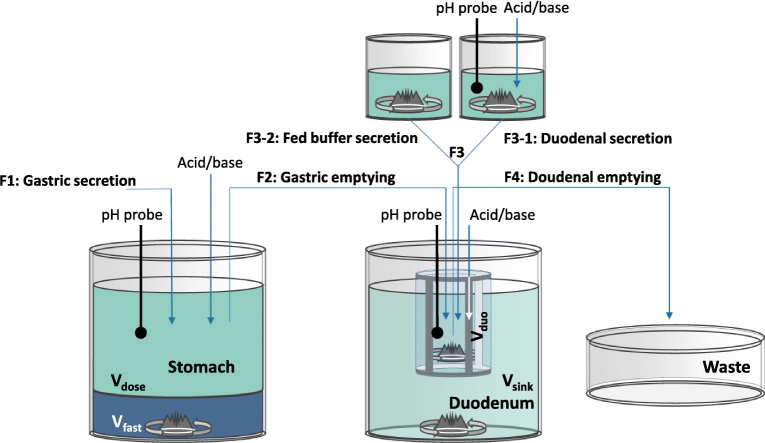

## 1 Introduction

*In vivo*-predictive *in vitro* models are very valuable tools to evaluate the gastrointestinal behavior of oral dosage forms thereby accelerating the formulation development process and de-risking drug development projects (Butler et al., 2019; Vinarov et al., 2021). Upon administration, the absorption of a drug compound into the systemic circulation is dependent on many physically and dynamically interconnected processes such as the disintegration of the dosage form, the release of the drug, gastrointestinal dissolution, solubilization, precipitation, degradation, and transit of the released compound and its final permeation across the intestinal wall (Hens et al., 2021). The gastrointestinal tract is a complex environment which is characterized by specific dynamically changing intragastric and intestinal volumes and pH conditions (Koziolek et al., 2014, Koziolek et al., 2015a, Koziolek et al., 2015b; Mudie et al., 2014), compositions of gastric and intestinal juices (Kalantzi et al., 2006; Riethorst et al., 2016), gastrointestinal transfer (Hens et al., 2014), concentrations of food induced bile salt/phospholipid ratios (Beeckmans et al., 2018; Riethorst et al., 2016), and secretion of pancreatic enzymes (Keller and Layer, 2005) all of which differ in between fasted and fed state conditions and are dynamically changing in function of time during passage of the drug through the gastrointestinal tract. Depending on the physicochemical characteristics of the drug and its formulation the dynamic interconnection between these physiological processes can have a big impact on its behavior in the gastrointestinal tract (Dahan et al., 2016). Hence, for certain drug candidates the use of static dissolution tests can result in poor *in vivo*-predictive power and more complex *in vitro* gastrointestinal transfer models, that simultaneously simulate multiple factors, are required to provide pharmaceutical scientists with more *in vivo*-predictive data about the gastrointestinal behavior of oral dosage forms (Butler et al., 2019; Reppas et al., 2014; Vinarov et al., 2021).

Furthermore, many new drug candidates that emerge from modern drug discovery pipelines are characterized by an intrinsic very low aqueous solubility but adequate intestinal permeability (BCS class II drugs) thereby making their oral absorption solubility or dissolution rate-limited (Boyd et al., 2019; Buckley et al., 2013; Vinarov et al., 2021). The use of solubility-enabling approaches based on surfactants (Yang et al., 2018), cyclodextrins (Brewster et al., 2008), lipids (Porter et al., 2007), co-solvents (Williams et al., 2013), or amorphous solid dispersions (Schittny et al., 2020) or the concomitant intake of food have been used as strategies to increase the gastrointestinal solubility and dissolution rate of these drugs. However, increasing the intestinal solubility of drugs does not always translate into increased permeation, which can be attributed to the occurrence of a “solubility-permeability interplay” (Dahan et al., 2010, Dahan et al., 2016). Upon arrival in the small intestine, the above-mentioned strategies result in dissolved drug that is present in various colloidally associated or complexed states such as micelles, mixed micelles and vesicles formed by formulation additives (Raina et al., 2015; Boyd et al., 2019). However, these colloidal states can not cross the intestinal barrier and to become available for absorption the drug must be released from these complexes and be present as molecularly (free) dissolved drug (Berben et al., 2017, Berben et al., 2018b; Buckley et al., 2013; Frank et al., 2012; Hens et al., 2015; Holmstock et al., 2013). Determination of the intestinal solubility of a drug in *in vitro* dissolution tests only generates information about the apparent solubility, a combination of both the colloidal-associated and molecularly dissolved drug, possibly overestimating the effect of the formulation strategy on absorption (Berben et al., 2018a; Borbás et al., 2019). Thus, to make convincing statements about formulation strategies and dosing regimens on the bioaccessibility of a drug, it is necessary to discriminate between the apparent and molecular solubility which can be achieved by the assessment of drug permeation together with dissolution testing (Berben et al., 2017, Berben et al., 2018a; Boyd et al., 2019; Raina et al., 2015; Vinarov et al., 2021). Multiple permeation devices exist differing in their general geometry, permeation barrier used, donor and acceptor volumes, and permeation surface area which can be subdivided in off-line permeation tools and set ups that combine dissolution and permeation (Berben et al., 2018a; Buckley et al., 2012). However, these permeation models are mainly static, using snap-shot intestinal media with a constant composition and pH and do not simulate gastric and duodenal emptying and duodenal secretions. This results in a constant high concentration of the total drug (solid and dissolved), formulation additives, and food-induced secretions in the donor compartment of the system (Berben et al., 2018a; Kostewicz et al., 2014). *In vivo*, oral drug delivery systems are exposed to rapidly changing gastrointestinal conditions both in the stomach and small intestine and the interplay between gastric secretions, gastric emptying, duodenal secretions, and duodenal emptying results in dynamic concentration profiles of the drug in the intestinal lumen. Hence, drug molecules are continuously exchanged between their colloid-associated and molecularly dissolved states. Furthermore, once molecularly dissolved the drug is removed from the lumen through passive transcellular diffusion (BCS class II drugs) and this during physiologically relevant time periods which corresponds with its gastrointestinal transit (Boyd et al., 2019; Dahan et al., 2016). For these reasons, the dynamically changing gastrointestinal environment and the physically interconnected kinetics of gastrointestinal drug dissolution and intestinal permeation are crucial features of *in vitro* tools for generating *in vivo*-predictive data about the gastrointestinal behavior of drugs and their formulations (Berben et al., 2018a; Vinarov et al., 2021). Numerous critical parameters and features need to be considered when developing a permeation carrier to accurately simulate the dynamics of interconnected dissolution and permeation. First, the donor compartment of the permeation carrier should simulate the dynamic small intestinal lumen and be surrounded by a semi-permeable membrane. The permeation carrier needs to be in contact with an acceptor solution that contains adequate sink conditions. The structure and general geometry of the permeation carrier should allow the simulation of gastric emptying, duodenal secretions, and duodenal emptying in the donor compartment to generate biorelevant concentration profiles of a drug in the donor compartment (Hens et al., 2014). In addition, active pH control is necessary for the study of the gastrointestinal dissolution and intestinal permeation of ionizable drugs for which the pH shift during transfer from stomach to duodenum dictates their dissolution (Buckley et al., 2012; Kostewicz et al., 2004; Kourentas et al., 2016; Rubbens et al., 2016). Adequate mixing of both the donor and acceptor compartment is required to reduce the unstirred water layer and to ensure reliable permeability assessment (Berben et al., 2018a; Buckley et al., 2012). The donor volume of the carrier should contain a volume that is biorelevant for the duodenum thereby resulting in biorelevant duodenal drug concentration profiles (Hens et al., 2014). The permeation area to donor volume ratio should be maximized to allow proper sink conditions and should preferably be around 2 cm^−1^ (Berben et al., 2018a; Vinarov et al., 2021). The carrier should be mounted in a dynamic gastrointestinal transfer system and sampling of both the stomach compartment, duodenal donor compartment, and duodenal acceptor compartment should be possible thereby generating mechanistic insights into the gastrointestinal dissolution, solubilization, supersaturation, precipitation, and permeation of an oral drug.

The aim of the present study was to develop a dynamic gastrointestinal transfer model with physically interconnected permeation, namely the Diamod® and to validate its *in vivo*-predictive power by studying the influence of concomitant water intake on the gastrointestinal behavior of itraconazole, formulated as a Sporanox® solution, and the influence of dosing indinavir sulfate under fasted or fed state conditions on its gastrointestinal dissolution and permeation. Both case studies were selected based on the availability of *in vivo* data that clearly demonstrated that the transfer from stomach to duodenum had a big impact on the duodenal solubility of the drugs (both itraconazole and indinavir sulfate are weak basic drugs) and that the plasma concentrations of the drugs were strongly influenced by the occurrence of a solubility-permeability interplay thereby necessitating the use of an *in vitro* model that simulates both the dynamic gastrointestinal transfer and physically and kinetically interconnected permeation of a drug substance during passage through the gastrointestinal tract (Berben et al., 2017; Brouwers et al., 2017; Carver et al., 1999; Holmstock et al., 2013; Rubbens et al., 2016).

## 2 Materials & methods

### 2.1 Chemicals

Indinavir (USP reference standard) and itraconazole were purchased from Merck Life Science B.V. (Overijse, Belgium) while indinavir sulfate was acquired from Abcam (Cambridge, United Kingdom). Sporanox® (10 mg/mL itraconazole) was bought at a local pharmacy. Oxgall was obtained from BD Bioscience (Aalst, Belgium) and lecithin was ordered from Carl Roth (Karlsruhe, Germany). Sodium chloride (NaCl), pepsin, sodium hydrogen carbonate (NaHCO_3_), potassium chloride (KCl), magnesium sulfate hepta-hydrate (MgSO_4_.7H_2_O), and potassium di‑hydrogen phosphate (KH_2_PO_4_) were acquired from Chem-lab analytical BVBA (Zedelgem, Belgium). Gastric lipase, pancreatin, HEPES (1 M) as well as D-α-tocopheryl polyethylene glycol 1000 succinate (TPGS) were bought from Merck Life Science B.V. (Overijse, Belgium). Sodium hydroxide (NaOH), calcium di-chloride (CaCl_2_), di‑sodium hydrogen phosphate di-hydrate (Na_2_HPO_4_.2H_2_O), glucose, methanol (HPLC-gradient grade), acetonitrile (ACN, LC-MS grade), and high purity water for HPLC were all purchased from VWR International Europe BVBA (Leuven, Belgium). SnakeSkin™ regenerated cellulose membranes with a molecular weight cut-off of 3500 Da and an inner diameter of 35 mm were also ordered from VWR International Europe BVBA. Water for media and experimental runs was purified using an Elix Advantage 10 water purification system (Merck Millipore, Darmstadt, Germany) while water for UHPLC analysis and sample preparation was purified using a Milli-Q® IQ 7010 system (Merck Millipore) equipped with a Milli-Q® Q-pod (0.22 μM filter).

### 2.2 Media

The sink medium, used in the acceptor compartment of the duodenum, was based on Hank's balanced salt solution (HBSS) (8 g/L NaCl, 0.4 g/L KCl, 0.14 g/L CaCl_2_, 0.2 g/L MgSO_4_.7H_2_O, 0.06 g/L Na_2_HPO_4_.2H_2_O, 0.06 g/L KH_2_PO_4_, 1 g/L glucose and 0.35 g/L NaHCO_3_). Glucose, D-α-tocopheryl polyethylene glycol 1000 succinate (TPGS), and HEPES were added to this solution to reach a final concentration of 0.025 M, 10 g/L, and 10 mM, respectively. The pH was adjusted to 6.5 (fasted state simulations) or 5.8 (fed state simulations) using 2 M NaOH and 5 M HCl. Simulated gastric juice and gastric secretions were based on a previously described recipe of FaSSGF (Jantratid and Dressman, 2009) and were prepared by dissolving 0.3 g/L oxgall, 0.02 g/L lecithin, 11.08 g/L NaCl, 1.58 g/L pepsin, and 9.6 g/L gastric lipase (only added for fed experiments) in pure water; the pH was adjusted to 1.6. Duodenal secretions were based on FaSSIF-V2 (Jantratid and Dressman, 2009) and were prepared as follows for the fasted state: 12.25 g/L KH_2_PO_4_, 4.11 g/L oxgall, 2.78 g/L NaOH, 8.02 g/L NaCl, and 5.35 g/L of pancreatin were dissolved in pure water and the pH was adjusted to 7.9. The fed state duodenal secretions were prepared by dissolving 10.11 g/L KH_2_PO_4_, 17.48 g/L oxgall, 2.3 g/L NaOH, 6.62 g/L NaCl, and 22.38 g/L pancreatin in purified water after which the pH was adjusted to 5.8. Fasted and fed state simulated duodenal juice were obtained by diluting the fasted state duodenal secretions two times in pure water and adjusting the pH to 6.5 and 5.8, respectively. For fed state experiments a buffer solution was also prepared by adding 10.11 g/L KH_2_PO_4_, 2.3 g/L NaOH, and 6.62 g/L NaCl to pure water, pH was adjusted to pH 5.8. The fed state carbohydrate solution was obtained by dissolving 375 g/L glucose in pure water after which the pH was adjusted to 1.6. For the fed state experiments simulating low gall bladder activity, a reduced duodenal simulation fluid was prepared by dissolving 10.11 g/L KH_2_PO_4_, 3.43 g/L oxgall, 2.3 g/L NaOH, 6.62 g/L NaCl, and 22.38 g/L pancreatin in purified water after which the pH was adjusted to 6.5.

### 2.3 Diamod® experiments

#### 2.3.1 Diamod® general features

**Fig. 1:**
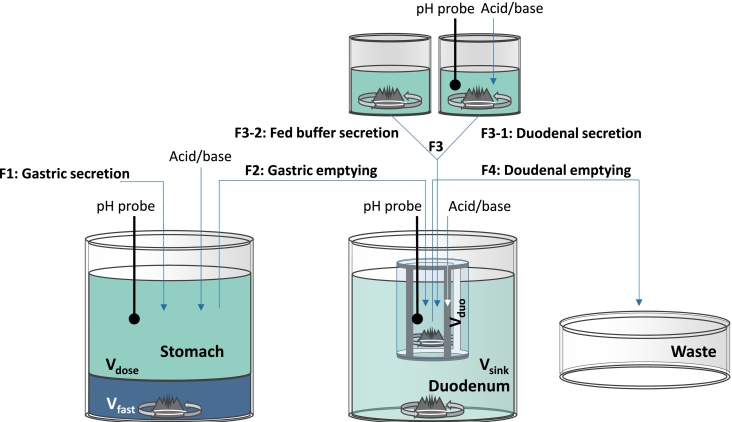
Schematic illustration of the Diamod®, a dynamic *in vitro* gastrointestinal transfer system with physically interconnected permeation.

The Diamod® is an automated computer-controlled dynamic gastrointestinal transfer model with interconnected permeation consisting of 2 reactor vessels (Fig. 1). The first vessel, simulating the stomach, consists of a custom-made double jacket glass reactor (95 mm internal diameter for fed state, 75 mm internal diameter for fasted state; ProDigest B.V., Zwijnaarde, Belgium), allowing temperature control through the use of a circulating water bath, which is sealed at the top with a custom-made lid (ProDigest B.V.) that contains dedicated passageways to allow the positioning of pH electrodes (Mecotrode Flat; Hamilton GmbH, Höchst im Odenwald, Germany), tubing (CellGyn TPE tubing, internal diameter 3.2 mm; Watson-Marlow N.V., Zwijnaarde, Belgium) to allow gastric secretions into the stomach and tubes that allow gastric emptying of the stomach (E Tygon tubing, internal diameter 2.79 mm; Metrohm Belgium NV, Antwerpen, Belgium), tubing (CellGyn TPE tubing, internal diameter 3.2 mm; Watson-Marlow N.V.) for automatic pH control, and ports for sampling of the stomach reactor (Silicone tubing, internal diameter 2 mm; VWR International Europe BVBA). The vessel is put on top of a magnetic stirrer (MIX 1, 2mag AG, Munich, Germany) that drives a magnetic stirring bar positioned in the fasted state stomach reactor (Pivot ring stirrer bar, 30 × 6 mm, PTFE; VWR International Europe BVBA) or fed state stomach reactor (Equilateral triangular with 25.5 mm sides, 40 mm long, PTFE; Chemlab-Analytical BVBA, Zedelgem, Belgium). The stomach vessel accepts gastric secretions (Flow rate F1) and is continuously emptied (Flow rate F2) into the second reactor vessel which simulates the lumen of the duodenum and the blood stream that accepts the permeated fraction. The second vessel consists of an inner donor compartment that is mounted inside an outer acceptor compartment. The acceptor compartment consists of a custom-made double jacket glass reactor (75 mm internal diameter; ProDigest B.V.), allowing to control the temperature of its content through the use of a circulating water bath. The acceptor compartment simulates the blood stream and contains a sink solution to accept the test compound that permeates from the inner donor compartment. The inner donor compartment, simulating the duodenal lumen, consists of a custom-made cylindrical grid structure that can support a 35 mm internal diameter semi-permeable membrane (ProDigest B.V.). A semi-permeable regenerated cellulose membrane (Snakeskin™) is mounted around this grid structure with a molecular weight cut-off of 3500 Da. Hence, the inside of this grid structure serves as the donor compartment of the permeation device and the sink solution as the acceptor compartment of the permeation device. Molecularly dissolved drug in the inside of the donor compartment can immediately permeate over the regenerated cellulose membrane into the acceptor compartment resulting into a physically and dynamically interconnected dissolution and associated permeation process. The geometry of the inner donor compartment is designed to contain biorelevant duodenal volumes (30 mL), to generate a permeation surface area of 65 cm^2^ and to result in a permeation surface area to donor volume ratio of 2 cm^−1^. A rotating magnetic field (MIX 1, 2mag AG) drives two stirrers which homogenize fluids both in the donor (Equilateral triangular stirrer with 6 mm sides, 12 mm long, PTFE; VWR International Europe BVBA) and acceptor (Equilateral triangular stirrer with 25.5 mm sides, 40 mm long, PTFE; Chemlab-Analytical BVBA) compartments. The second vessel is sealed at the top with a lid that contains multiple passageways that serve to allow gastric emptying of the stomach inside the donor compartment of the second vessel, allow positioning of tubing (CellGyn TPE tubing, internal diameter 3.2 mm; Watson-Marlow N.V.) for duodenal secretions (Flow rate F3–1) and fed buffer (Flow rate F3–2) secretions from an external reservoir inside the donor compartment of the second vessel, and positioning of tubing (E Tygon tubing, internal diameter 2.79 mm; Metrohm Belgium NV) for emptying (Flow rate F4 = F2 + F3–1 + F3–2) of the donor compartment of the second vessel into a waste container. All fluid transfers are mediated by peristaltic precision pumps (combination of Watson-Marlow and Ismatec pumps), controlled by a custom-made software (ProDigest B.V.). Furthermore, the lid is designed in such a way that a pH electrode can be mounted in the inside of the inner donor compartment, allowing pH control, and the presence of sampling ports that allow to take samples from both the donor and acceptor compartment of the second vessel.

It is mandatory that *in vitro* dynamic gastrointestinal transfer models adequately simulate the dynamics of gastric emptying, gastric secretions, duodenal secretions, and duodenal emptying to accurately simulate biorelevant drug concentrations during transit through the gastrointestinal tract. During a small scale clinical study Hens et al. (2014) determined the intraluminal concentrations of a marker molecule, namely paromomycin, in aspiration samples (stomach and duodenum) taken under fasted state and fed state conditions. Since paromomycin has a high aqueous solubility and is not absorbed through the small intestinal epithelium, the intraluminal concentrations of this compound are only depending on the gastrointestinal volumes and the dynamics of gastric emptying, gastric secretions, duodenal dilution, and duodenal emptying. Hence, these data were used as a reference to optimize the different flow rates of the system. A mathematical model was constructed for the Diamod® that allowed to theoretically calculate the total concentration of a drug substance in the stomach and the duodenum in function of time. Subsequently, the theoretical values generated by this mathematical model were compared to the *in vivo*-observed intraluminal concentrations of paromomycin in the duodenum under fasted and fed state conditions. Fitting of the theoretical concentrations with the *in vivo* data generated the necessary flow rates to be implemented in the Diamod® in order to obtain an accurate simulation of the drug concentration in function of time as observed *in vivo*. In the study of Hens et al. (2014) the obtained *in vivo* data were also used as a reference thereby demonstrating that several parameters associated with gastric emptying and duodenal secretions used in other *in vitro* models needed to be adapted to obtain biorelevant concentration curves of the drug.

Following the optimization of the gastrointestinal volume and flow rate parameters of the Diamod® the concentrations of enzymes and bile salts present in the Diamod® were determined using the mathematical model. Based on *in vivo* data generated about fasted and fed state stomach pepsin activities (Jantratid and Dressman, 2009, Kalantzi et al., 2006; Minekus et al., 2014), duodenal bile salt concentrations (Beeckmans et al., 2018; Riethorst et al., 2016), and duodenal enzymatic activities (Keller and Layer, 2005) the compositions of the simulatory media were determined in order to reflect the *in vivo*-observed conditions.

A regenerated cellulose membrane with a molecular weight cut-off of 3500 Da was used in the Diamod®. Holmstock et al. (2013) performed static permeation experiments to discriminate between free moleculary dissolved indinavir and colloidal-associated indinavir (entrapped in mixed micelles under fed state conditions) using regenerated cellulose membranes with a molecular weight cut-off of 12–14 kDa (pore size ≈ 3 nm). This membrane was expected to be impermeable for micelles since these micelles were shown to be about 7 nm and 50 nm in diameter in FeSSIF and FaSSIF, respectively. However, a smaller molecular weight cut-off of the membrane is necessary to study the solubility-permeability interplay of itraconazole formulated with 2-hydroxypropyl-β-cyclodextrin (2-HP-β-CD). The *in vivo*-observed solubility-permeability interplay has been attributed to the non-linear dependence between the itraconazole solubility and the 2-HP-β-CD concentration which indicates that higher order complexes are formed at increasing cyclodextrin concentrations (Berben et al., 2017). The formation of these higher order complexes (1 molecule of itraconazole complexed with two or three molecules of 2-HP-β-CD) will result in colloidal species that will be retained by a regenerated cellulose membrane with a molecular weight cut-off of 3500 Da.

#### 2.3.2 Diamod®: Intraluminal dilution of Sporanox®

Sporanox® is a cyclodextrin-based solution (40% 2-hydroxypropyl-β-cyclodextrin; 2-HP-β-CD) of the lipophilic (clogP 6.2) and weakly basic (pKa 2.0 and 3.7) drug itraconazole. Considering the non-linear dependence between 2-HP-β-CD concentrations and itraconazole solubility Berben et al. (2017) performed a clinical study to determine the effect of intraluminal dilution of the cyclodextrin-based solution on the gastrointestinal behavior of itraconazole. To do so, 20 mL of a Sporanox® solution was administered to fasted state healthy volunteers without water intake or with the co-administration of 240 mL of water. Determination of the concentration of itraconazole in gastrointestinal aspiration and plasma samples revealed that the intake of water significantly decreased the duodenal solubility of itraconazole whereas plasma levels of itraconazole were unaffected by the dosing regimen. These observations were attributed to the occurrence of the solubility-permeability interplay in the intestine (Berben et al., 2017, 2018b; Brouwers et al., 2017). Hence, in order to investigate the *in vivo*-predictive simulatory power of the Diamod®, the influence of concomitant water intake on the gastrointestinal behavior of itraconazole, formulated as a Sporanox® solution and taken under fasted state conditions was investigated in the Diamod® system.

Before the start of the experiments, 55 mL of simulated gastric juice (V_fast_ = 55 mL) was added to the first compartment (stomach vessel) to simulate the basal volume of the stomach under fasted conditions. Next to this, 30 mL of fasted simulated duodenal juice (V_duo_ = 30 mL) was added to the inner compartment of the second vessel (simulation duodenal lumen) and 300 mL of sink solution (V_sink_ = 300 mL) was added to the outer compartment of the second vessel (simulation of the blood stream). At the start of the first experimental run 20 mL of Sporanox® solution (Janssen, Beerse, Belgium), corresponding to a dose of 200 mg itraconazole, was added to the stomach (without water; V_dose-1_ = 20 mL) whereas at the start of the second experiment, 20 mL of Sporanox® solution was added together with 240 mL of water (with water; V_dose-2_ = 260 mL). Following administration of the dose the dynamics of the gastrointestinal tract were simulated through the administration of gastric secretions (F1 = 2.5 mL/min) to the stomach compartment. Simultaneously, the content of the stomach was emptied into the duodenum using flow rate F2 which is a combination of a first order kinetics gastric emptying of V_dose_ with a half time of gastric emptying (T_1/2_) of 19 min and a linear emptying of the stomach to compensate for the volume of added gastric secretions to the stomach. The stomach was emptied inside the inner donor compartment of the second vessel. Fasted state gall bladder and pancreatic activity was simulated by a constant flow rate of fasted state duodenal secretions from an exterior vessel inside the inner donor compartment of the second vessel (F3–1 = 2.5 mL/min). The content of the duodenum was continuously emptied throughout the experiment (Flow rate F4 = F2 + F3–1). Hence, the volume of the stomach decreased monoexponentially in function of time whereas the volume of the inner donor and the outer acceptor compartment of the second vessel remained constant throughout the run. The pH of the content of the stomach was automatically controlled at a setpoint of 1.6 by addition of 0.5 M of NaOH and HCl. The pH of the lumen of the duodenum (donor compartment of the second vessel) was kept constant at a value of 6.5 through active pH control (addition of 0.5 M of NaOH and HCl) and the secretion of duodenal fluids from the exterior reservoir inside the donor compartment of the second vessel. Active pH control was also applied to the duodenal secretions inside the exterior reservoir to change the buffering capacity of the duodenal secretions in function of time through the addition of 2 M NaOH and 3.5 M HCl. This was mandatory since during the initial stages of the run high flow rates of acidic gastric content empty in the duodenum whereas during later stages the flow rate of acidic gastric content from the stomach decreases.

The content of the stomach reactor was homogenized through stirring at 300 rpm. The content of the inner (lumen of duodenum) and outer (bloodstream) compartment of the second vessel was homogenized through stirring at 500 rpm. The temperature of the compartments simulating the stomach, duodenum, and blood stream was controlled at 37 °C.

Each experimental run was conducted over an 180 min period and samples (1 mL) were taken from the stomach, duodenal lumen, and permeated fraction after 7, 15, 30, 45, 60, 75, 90, 105, 120, 135, 150, 165, and 180 min. After sampling, 1 mL of fresh medium was added to the respective reactors. Both the total and solute concentration of itraconazole was determined in the samples taken from the stomach and duodenum. Total concentrations were determined by preparing a 40-fold dilution of the stomach and duodenal samples in a methanol/water mixture (50:50; v/v %). Afterwards, the samples were centrifuged (18,213 x*g*, 7 min, 37 °C) and the supernatant was used for UHPLC-PDA analysis (see section 2.4). For determination of solute concentrations, samples were centrifuged (18,213 x*g*, 7 min, 37 °C) and the supernatant was diluted 40- or 20-fold in a methanol/water mixture (50:50; v/v %). Afterwards, the diluted samples were centrifuged (18,213 x*g*, 7 min, 37 °C) and the recovered supernatant was used for UHPLC-PDA analysis (see section 2.4). Samples taken from the sink solution (permeated fraction) were diluted two-fold in a mixture of methanol/water (50:50; v/v %). Afterwards, the diluted samples were centrifuged (18,213 x*g*, 7 min, 37 °C) and the supernatant was used for UHPLC-PDA analysis (see section 2.4).

Next to this, the thermodynamic solubility of itraconazole in all the samples taken from the duodenal lumen was determined. Sample preparation involved the addition of an excess of pure itraconazole to samples taken from the duodenum following incubation of the samples in a shaking incubator for 24 h at 37 °C. Following incubation, samples were centrifuged (18,213 x*g*, 7 min, 37 °C) and the supernatant was diluted 20-fold in a mixture of methanol/water (50:50; v/v %). Afterwards, the samples were centrifuged (18,213 x*g*, 7 min, 37 °C) and the supernatant was used for UHPLC-PDA analysis (see section 2.4). Based on the values of the thermodynamic solubility, the degree of supersaturation (DS) in each of the duodenal samples was determined by dividing the duodenal solute concentrations with the thermodynamic solubility values as determined in these samples. All experiments were performed in biological triplicate.

#### 2.3.3 Diamod®: Negative food effect for indinavir sulfate

Indinavir, an HIV protease inhibitor, is a weak basic compound with pKa's of 3.7 and 5.9, having a very high solubility under acidic conditions (> 162 mM at pH < 3.5) whereas its solubility under neutral conditions (50 μM at pH 6) is limited (Carver et al., 1999; Rubbens et al., 2016). To achieve sufficient plasma levels a solid oral dose of indinavir is administered as a sulfate salt instead of the free base. Yeh et al. (1998) demonstrated that indinavir sulfate is subjected to a negative food effect when administered together with a high-fat, high-caloric breakfast which could have been caused by the elevated gastric pH and delayed gastric emptying under fed state conditions compared to the low gastric pH and fast gastric emptying present under fasted state conditions. To test these hypotheses, Carver et al. (1999) performed a small clinical study during which indinavir sulfate was administered to fasted state individuals and fed state individuals consuming isocaloric meals with different compositions. Gastric pH was measured along indinavir plasma concentrations. The consumption of a high caloric protein meal resulted in a substantial increase in stomach pH as compared to the fasted stomach pH, thereby resulting in a reduction of 68% in systemic exposure. Consumption of an isocaloric carbohydrate meal (similar effect on gastric emptying kinetics as the protein meal) had no substantial effect on the stomach pH as compared to fasted state conditions. Nevertheless, the consumption of this carbohydrate meal resulted in a decrease of 45% in systemic exposure relative to fasted state conditions. Thus, these data demonstrated that the elevated gastric pH under fed state conditions, impacting the pH-dependent indinavir dissolution, is not the only factor contributing to the observed negative food effect. Indeed, consumption of food results in the presence of high concentrations of bile salts, phospholipids, and digestion products in the duodenum which can have substantial effects on the solubility and permeability of a drug. Holmstock et al. (2013) demonstrated that the solubility of indinavir sulfate was 6-fold higher in fed state human intestinal fluids (FeHIF) as compared to fasted state HIF (FaHIF). However, the intestinal permeability of indinavir sulfate in FeHIF was 22-fold lower as compared to FaHIF. Hence, micellar entrapment of indinavir sulfate in the duodenum under fed state conditions can also contribute to the *in vivo-*observed negative food effect for indinavir sulfate. Considering these interesting findings, indinavir sulfate was tested in the Diamod® to generate mechanistic insights into the dynamic interplay between the effects of gastric pH, gastric emptying, and micellar entrapment on the gastrointestinal dissolution and intestinal permeation of indinavir under fasted and fed state conditions.

Before the start of the fasted state experiments, 55 mL of simulated gastric juice (V_fast_ = 55 mL) was added to the stomach. For the duodenal compartment, 30 mL of simulated duodenal juice (V_duo_ = 30 mL) was added to the inner compartment, simulating the lumen of the duodenum, and 300 mL of a sink solution (V_sink_ = 300 mL) was added to the outer compartment, simulating the bloodstream. At the start of the experiments, 696 mg of indinavir sulfate, corresponding to a dose of 600 mg indinavir, was added to the stomach together with 250 mL of water (V_dose_ = 250 mL). Upon dosing, the system started to simulate the dynamics of the fasted state gastrointestinal tract. This was done by continuous addition of gastric secretions to the stomach (Flow rate F1 = 2.5 mL/min) and continuous gastric emptying (Flow rate F2) which is a combination of a first order kinetics gastric emptying of V_dose_ with a half time of gastric emptying (T_1/2_) of 19 min and a linear emptying of the stomach to compensate for the volume of added gastric secretions to the stomach. Fasted state bile salt concentrations and enzymes were simulated by the addition of duodenal secretions to the lumen of the duodenum (Flow rate F3–1 = 2.5 mL/min). The content of the duodenum was continuously emptied throughout the experiment (Flow rate F4 = F2 + F3–1). Thus, the volume of the stomach decreased monoexponentially in function of time whereas the volume of the duodenum (inner compartment) and sink solution (outer compartment) remained constant. The pH of the content of the stomach was automatically controlled at a setpoint of 1.6 by addition of 0.5 M of NaOH and HCl. The pH of the duodenum (inner compartment) was controlled at a setpoint of 6.5 through active pH control (addition of 0.5 M of NaOH and HCl) and through the addition of duodenal secretions. The pH of the duodenal secretions was controlled in function of time at dedicated setpoints, through the addition of 2 M NaOH and 3.5 M HCl, to generate enough buffer capacity to keep the duodenum at a pH value of 6.5.

The content of the stomach reactor was homogenized through stirring at 300 rpm. The content of the duodenal vessel and sink vessel was homogenized through stirring at 500 rpm. The temperature of all vessels was controlled at 37 °C.

Each experimental run was followed for 240 min and samples (1 mL) were taken from the stomach vessel, duodenal lumen, and permeated fraction after 7, 15, 30, 45, 60, 75, 90, 105, 120, 135, 150, 165, 180, 195, 210, 225, and 240 min. After sampling, 1 mL of fresh medium was added to the respective vessels. Total concentrations of indinavir in the stomach, duodenal lumen, and permeated fraction were determined by diluting the sample 20-, 40-, or 6-fold in a mixture of methanol/water (50:50; v/v %), respectively. Afterwards, dilutions were centrifuged (18,213 x*g,* 7 min, 37 °C) and the supernatant was used for UHPLC-PDA analysis (see section 2.4). Solute concentrations of indinavir were determined in the stomach and duodenal lumen by initial centrifugation of the samples (18,213 x*g*, 7 min, 37 °C). Afterwards, the supernatant was diluted 6-fold in a mixture of methanol/water (50:50; v/v %), following centrifugation (18,213 x*g*, 7 min, 37 °C). The resulting supernatant was used for UHPLC-PDA analysis (see section 2.4). All fasted state experiments were performed in biological triplicate.

Three sets of fed state experiments were performed. Before the start of the experiments the same volumes of simulated fasted gastric fluid, simulated fasted duodenal fluid, and sink solution were added to the respective vessels. At the start of the experiments, simulating high stomach pH conditions (protein meal; Carver et al., 1999), 696 mg indinavir sulfate was added to the stomach together with 400 mL of the nutritional drink Ensure® plus (Abbott Laboratories, B.V., Zwolle, The Netherlands) and 250 mL of water. At the start of the low stomach pH fed experiments (carbohydrate meal; Carver et al., 1999), 696 mg of indinavir sulfate was added to the stomach together with 400 mL of a glucose-based carbohydrate solution (same caloric value as 400 mL Ensure® plus) and 250 mL of water. After 15 min, simulating the lag phase for gastric emptying under fed state conditions, the dynamics of the fed state upper gastrointestinal tract were simulated through gastric secretions (Flow rate F1 = 2.5 mL/min) of simulated gastric fluids into the stomach and gastric emptying [Flow rate F2; combination of emptying of added gastric secretions (2.5 mL/min) and a flow rate (2.7 mL/min) to empty the added dose (650 mL) from the stomach in a 4-h period] from the stomach to the duodenum. The gastric secretion rate used during the fed state experiments in combination with the composition of the gastric secretion media resulted in biorelevant concentrations of pepsin in the stomach. The same high gastric secretion rate was used during the fasted state experiments (see above) to improve the operational efficiency and reproducibility of the Diamod®. Once V_dose_ is emptied through gastric monoexponential emptying under fasted state conditions, the content of the stomach is continuously transferred from the stomach to the duodenum at a flow rate that equals the gastric secretion rate. Since during this period of the experiment solid particles could still be present in the stomach a flow rate of 2.5 mL/min was chosen for fasted state conditions in order to be capable to transfer the solid particles through the action of peristaltic pumps. The added dose was emptied from the fed state stomach during a 4-h period. As mentioned above, the parameters of the Diamod® were based on the *in vivo* data of the intraluminal concentration of paromomycin under fed state conditions (Hens et al., 2014). During this *in vivo* trial samples were taken over a period of 4 h for experiments under fed state conditions, revealing that 4 h are sufficient to get a good overview of the concentration-time profiles of the drug of interest. Furthermore, Minekus et al. (2014) state that the half time of gastric emptying of a nutritious and semi-solid meal is 2 h. Therefore, the Diamod® stomach was emptied over a period of 4 h in the present study.

A constant duodenal secretion rate (F3 = 8 mL/min) was applied to the duodenal lumen. For the fed state experiments using Ensure® plus and the carbohydrate experiments, simulating active gallbladder contractions and pancreatic secretions, the constant flow rate F3 consisted of a combination of the dynamically changing flow rates of a buffer solution with flow rate F3–2 and simulated fed state duodenal secretions with a flow rate F3–1 which were pumped from two external reservoirs inside the duodenum. The ratios of the flow rates F3–1 and F3–2 were selected to generate the dynamic bile salt concentrations in the duodenum as observed *in vivo* (Beeckmans et al., 2018; Riethorst et al., 2016). Briefly, the duodenal total bile salt concentrations remained at 3 mM during the initial 15 min of the experimental runs. Afterwards, the total bile salt concentrations increased linearly till a level of 15.4 mM between 15 min and 25 min of the run. This high bile salts concentration was maintained for 40 min after which the concentration of bile salts linearly decreased till fasted state levels (3 mM) after 100 min of the experimental run. Afterwards, fasted state total bile salt concentrations were maintained constant throughout the remainder of the experimental run. For the fed state experiments simulating the ingestion of a carbohydrate meal and the presence of fasted duodenal levels of bile salts (3 mM) a reduced duodenal simulation fluid was added to the duodenum at a constant flow rate F3–1 of 8 mL/min. Finally, the duodenum was emptied with a constant flow rate F6 (sum of gastric emptying and duodenal secretions). Consequently, the volume of the stomach emptied linearly in function of time whereas the volume of the duodenal donor and acceptor compartment remained constant. During the high stomach pH experiments the pH value of the stomach was controlled online, through the addition of 0.5 M NaOH and HCl, and was subjected to a sigmoidal decrease from an initial value of 4.6 till a final value of 1.6 over 240 min, simulating the impact of a protein rich meal on the stomach pH. During the experiments under low stomach pH conditions (carbohydrate meal with either high or low bile salt secretions in the duodenum) the pH of the stomach was controlled at a constant value of 1.6, through the addition of 0.5 M NaOH and HCl. During each fed state experiment, the pH of the duodenum was kept constant at a setpoint of 5.8 through a combination of active pH control (addition of 0.5 M NaOH and 0.5 M HCl) and buffer secretions. The pH of the sink solution was set at a value of 5.8.

The content of the stomach reactor was homogenized through stirring at 300 rpm. The content of the duodenal vessel and sink vessel was homogenized through stirring at 500 rpm. The temperature of the respective vessels was controlled at 37 °C.

Each experimental run was followed for 240 min and samples (1 mL) were taken from the stomach, the duodenal lumen, and the permeated fraction after 7, 15, 30, 45, 60, 75, 90, 105, 120, 135, 150, 165, 180, 195, 210, 225, and 240 min. After sampling, 1 mL of fresh medium was added to the respective reactors. In the samples taken from the stomach and duodenal lumen both the total and solute concentration of indinavir were determined. Total concentrations were determined by preparing a 20- or 40-fold dilution in a mixture of methanol/water (50:50; v/v %). Afterwards, the dilutions were centrifuged (18,213 x*g*, 7 min, 37 °C) and the supernatant was used for UHPLC-PDA analysis (see section 2.4). Solute concentrations were determined by centrifugation of the samples (18,213 x*g*, 7 min, 37 °C) and diluting the supernatant 6- or 20-fold in a mixture of methanol/water (50:50; v/v %). Afterwards, the dilutions were centrifuged (18,213 x*g*, 7 min, 37 °C) and the supernatant was used for UHPLC-PDA analysis (see section 2.4). In the samples taken from the permeated fraction (acceptor compartment) the total concentration of indinavir was determined by initially diluting the samples two-fold in a mixture of methanol/water (50:50; v/v %). Afterwards, the samples were centrifuged (18,213 x*g*, 7 min, 37 °C) and the supernatant was used for UHPLC-PDA analysis (see section 2.4).

Moreover, the thermodynamic solubility of indinavir in all the samples taken from the lumen of the duodenum was determined. Sample preparation involved the addition of an excess of pure indinavir to samples taken from the duodenum following incubation of the samples in a shaking incubator for 24 h at 37 °C. Following incubation, samples were centrifuged (18,213 x*g*, 7 min, 37 °C) and the supernatant was diluted 20-fold in a mixture of methanol/water (50:50; v/v %). Afterwards, the dilutions were centrifuged (18,213 x*g*, 7 min, 37 °C) and the supernatant was used for UHPLC-PDA analysis (see section 2.4). Based on the values of the thermodynamic solubility, the degree of supersaturation (DS) in each of the duodenal samples was determined by dividing the duodenal solute concentrations with the thermodynamic solubility values as determined in these samples. All experiments were performed in biological triplicate.

### 2.4 UHPLC-PDA analysis

An Acquity®Arc™ with a 2998 PDA detector (Waters, Milford, MA, USA) was used as reverse-phase ultra-high performance liquid chromatography (RP-UHPLC) system to analyse the samples. Separation was performed using an XSelect CSH C18 XP Column (pore size 130 Å, particle size 2.5 μm, 3 mm i.d. x 100 mm, Waters, Milford, MA, USA) and an XSelect CSH C18 XP Vanguard Cartridge pre-column (pore size 130 Å, particle size 2.5 μm, 2.1 mm i.d. x 5 mm, Waters N.V., Antwerp, Belgium) for itraconazole and an XSelect HSS C18 XP Column (pore size 100 Å, particle size 2.5 μm, 2.1 mm i.d. x 100 mm, Waters, Milford, MA, USA) with and XSelect HSS T3 XP Vanguard Cartridge pre-column (pore size 100 Å, particle size 2.5 μm, 2.1 mm i.d. x 5 mm, Waters N.V., Antwerp, Belgium) for indinavir. For itraconazole a gradient was used with a mobile phase that consisted of 25 mM acetic acid buffer pH 3.5 (eluent A) and methanol (eluent B) [time zero 20 (A):80 (B) v/v %, a linear gradient was set to 10 (A):90 (B) v/v % at 4 min, the column was rinsed for 1 min 10 (A):90 (B) v/v % and re-equilibrated (1.5 min) with the start concentration of the mobile phase 20 (A):80 (B) v/v %, total run time 6.5 min]. All analyses were performed using a flow rate of 0.8 mL/min, an injection volume of 10 μL, and column temperature of 30 °C. Itraconazole eluted after 2.4 min, and was detected at a wavelength of 265 nm. For indinavir, a gradient was used with a mobile phase that consisted of 0.1 (v/v %) formic acid in water (eluent A) and acetonitrile (eluent B) [time zero 80 (A):20 (B) v/v %, a linear gradient was set to 10 (A):90 (B) v/v % at 3 min, the column was rinsed for 0.5 min 10 (A):90 (B) v/v % and re-equilibrated (2.5 min) with the start concentration of the mobile phase 80 (A):20 (B) v/v %, total run time 6 min]. All analyses were performed using a flow rate of 0.6 mL/min, an injection volume of 20 μL, and column temperature of 30 °C. Indinavir eluted after 2.6 min and was detected at a wavelength of 260 nm. The obtained peaks were integrated using Empower Pro software. Quantification of the concentrations of itraconazole and indinavir was performed using external standards. The limit of quantification (LOQ) of itraconazole and indinavir were 24 nM and 50 nM, respectively.

## 3 Results & discussion

### 3.1 Effect of intraluminal dilution on the performance of a cyclodextrin-based solution

**Fig. 2:**
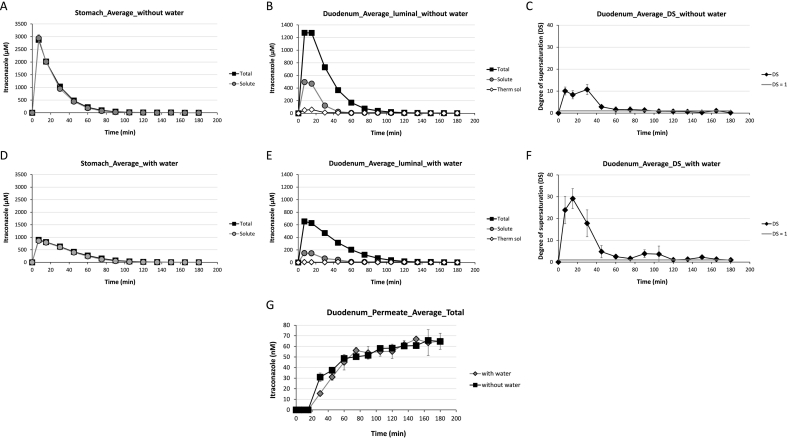
Average ± stdev (n = 3) concentration (μM) of total (black squares) and solute (grey circles) itraconazole in the stomach (A) and duodenum (B) of the Diamod®, thermodynamic solubility (white diamonds) of itraconazole in the duodenum (B) and degree of supersaturation (black diamonds) of itraconazole in the duodenum (C) upon administration of 20 mL Sporanox® solution without water. Average ± stdev (n = 3) concentration (μM) of total (black squares) and solute (grey circles) itraconazole in the stomach (D) and duodenum (E) vessel of the Diamod®, thermodynamic solubility (white diamonds) of itraconazole in the duodenum (E) and degree of supersaturation (black diamonds) of itraconazole in the duodenum (F) upon administration of 20 mL Sporanox® solution with water. Average ± stdev (n = 3) concentration (nM) of permeated itraconazole (G) upon administration of Sporanox® without water (black squares) and with water (grey diamonds).

To test the effect of intraluminal dilution on the performance of a Sporanox® solution in the Diamod®, two sets of experiments were executed. During the first experiment, 20 mL of Sporanox® solution was added to the fasted state stomach, containing 55 mL basal gastric juice, whereas during the second experiment 20 mL Sporanox® was added together with 240 mL of water. Sampling of the stomach compartment revealed that the total and solute concentrations of itraconazole were nearly identical in the stomach throughout the experiment and this independently of the intake of water (Fig. 2A and D). These data demonstrated that itraconazole remained completely dissolved during transit through the stomach in the Diamod®. Intake of water resulted in lower total and solute concentrations of itraconazole in the stomach as compared to intake without water hence resulting in the delivery of lower itraconazole concentrations to the duodenum. Indeed, intake of water resulted in a 1.9-fold decrease of the AUC_0-180min_ of solute itraconazole (Table 1). Comparison of the AUC_0-180min_ of total and solute itraconazole revealed that the lower concentrations of solute itraconazole were only due dilution with co-administered water and not due to the occurrence of precipitation events during the intake of water since the intake of additional water resulted in a comparable decrease in the total and solute AUC_0-180min_ of 45% and 47%, respectively (Table 1). Furthermore, the C_max_ of itraconazole in the stomach without water intake was 2940.4 ± 136.7 μM whereas it decreased to a concentration of 863.0 ± 8.6 μM upon the intake of water. These data correspond with the *in vivo* data generated by Berben et al. (2017) which demonstrated that itraconazole was completely dissolved in the stomach upon intake of Sporanox® and this independently of the co-administration of water. The intake of water did not result in precipitation effects but only in a dilution of the dosage form resulting in a 1.8 times reduction in the average of dissolved gastric AUC_0-3h_ of itraconazole (Berben et al., 2017). Furthermore, a clinical study performed by Brouwers et al. (2017) demonstrated that after intake of 20 mL Sporanox® solution together with 240 mL of water, itraconazole remained completely in solution in the stomach. Itraconazole has a solubility of 2.7 μM in FaSSGF (Bevernage et al., 2012). Hence, gastric concentrations of 2-HP-β-CD after intake of 20 mL Sporanox® together with 240 mL of water were capable to solubilize itraconazole and further stabilized the supersaturation of itraconazole in the stomach, since a degree of supersaturation in between 20 and 30 was observed for itraconazole in gastric aspiration samples (Brouwers et al., 2017). Stabilization of supersaturated itraconazole solutions by 2-HP-β-CD has also been demonstrated by Brewster et al. (2008).

**Table 1:** Stomach, duodenal, and permeated AUC_0-180min_ of total and solute itraconazole in the Diamod® upon administration of 20 mL Sporanox® solution to the stomach without and with the addition of 240 mL of water under fasted state simulatory conditions.

	Stomach		Duodenum		Permeate
	Average AUC_0-180min_ (μM*min) ± stdev		Average AUC_0-180min_ (μM*min) ± stdev		Average AUC_0-180min_ (nM*min) ± stdev
	Total	Solute	Total	Solute	Total
Sporanox® without water	73,601.0 ± 4046.3	70,658.6 ± 4631.9	45,544.5 ± 577.1	11,878.6 ± 242.0	8315.5 ± 219.9
Sporanox® with water	40,478.0 ± 612.1	37,434.3 ± 392.3	30,930.9 ± 433.3	5212.8 ± 122.0	8050.8 ± 708.9

Determination of the concentration of total and solute itraconazole revealed that itraconazole was not completely soluble in the duodenum (Fig. 2B and E). The transfer from the acidic stomach environment (pH 1.6) to the more neutral duodenal (pH 6.5) environment resulted in precipitation of itraconazole since the measured solute concentrations of itraconazole were lower than the total concentrations in the duodenal compartment of the Diamod®. Whereas the intake of water had no major effects on the solubility of itraconazole in the stomach, remarkable differences were observed in the duodenum. Notwithstanding the occurrence of precipitation, the intake of Sporanox® without water resulted in considerable concentrations of solute itraconazole, reaching a C_max_ of 495.3 ± 8.2 μM and an AUC_0-180min_ of solute itraconazole of 11,878.6 ± 242.0 μM*min. Intake of water had devastating effects on the solubility of itraconazole in the duodenum thereby lowering the C_max_ to 148.7 ± 18.1 μM and the AUC_0-180min_ to 5212.8 ± 122.0 μM*min (Table 1). Whereas the lower solute concentrations in the stomach upon water intake were clearly due to dilution of the dosage form with the co-ingested water the results obtained in the duodenum demonstrated that other factors contributed to this observation. Whereas water intake resulted in a reduction of the AUC_0-180min_ of the total concentration of itraconazole of 32.1% compared to intake without water, the AUC_0-180min_ for the solute concentration of itraconazole was decreased with 56.1% upon water intake (Table 1). Hence, the intake of water resulted in 24.0% more precipitation of itraconazole which was probably due to the intraluminal dilution of cyclodextrin in the duodenum thereby reducing the stabilization of supersaturated itraconazole. The duodenal behavior of itraconazole in the Diamod® is in line with the data obtained during clinical studies (Berben et al., 2017; Brouwers et al., 2017). In both studies substantial precipitation occurred when the weak base itraconazole was subjected to a pH shift upon transfer from the stomach into the duodenum. Berben et al. (2017) demonstrated that lower solute concentrations of itraconazole were present in the duodenum after intake of water as compared to the intake of Sporanox® without water and that this was not only due to a dilution effect of itraconazole but also due to a discrepancy in intestinal precipitation of itraconazole in between the two dosing regimens, as observed in the Diamod®. This effect was attributed to the intraluminal dilution of 2-HP-β-CD in the duodenum upon intake of water which probably lowered the itraconazole solubilizing properties in the duodenum. Indeed, during a follow-up study Berben et al. (2018c) determined the actual concentrations of 2-HP-β-CD in the duodenal aspirates of the volunteers. The authors demonstrated that, as previously observed in aqueous buffer media (Brewster et al., 2008), a non-linear relationship between itraconazole solubility and concentrations of 2-HP-β-CD existed in the duodenal aspirates of the subjects. Furthermore, intake of water resulted in a 2.3-fold decrease in the duodenal AUC_0-3h_ for 2-HP-β-CD, as compared to conditions without water intake. The high duodenal concentrations of 2-HP-β-CD in the duodenum upon Sporanox® administration without water resulted in the formation of higher order complexes between 2-HP-β-CD and itraconazole thereby resulting in less precipitation and increased solubilization of itraconazole upon transfer from the stomach to the duodenum. In the same study, Berben et al. (2017) observed that intake of water resulted in substantially lower concentrations of 2-HP-β-CD also in the duodenum thereby explaining the increased precipitation of itraconazole. Interestingly, determination of the degree of supersaturation in the duodenal samples of the Diamod® demonstrated that the concomitant intake of water resulted in higher degrees of supersaturation of itraconazole in the duodenum as compared to the conditions without water intake (Fig. 2C and F). This is in line with the above-mentioned clinical data and demonstrate that the solubilization, supersaturation, and precipitation of itraconazole was adequately simulated in the Diamod® system.

Because intake of water resulted in a decrease of the AUC_0-180min_ of solute itraconazole with 56.1% in the Diamod® it could be anticipated that the intake of water would result in a substantial reduction in permeation of itraconazole in the acceptor compartment. Determination of the concentrations of permeated itraconazole demonstrated that comparable concentrations of itraconazole were permeated in the acceptor compartment of the Diamod® (Fig. 2G). Indeed, the intake of water only resulted in a decrease of 3.2% in the AUC_0-180min_ of permeated drug (Table 1). Similar observations were made during the clinical study by Berben et al. (2017) and it was assumed that this discrepancy could be explained by differences in the extent of entrapment of itraconazole in the duodenum caused by differential complexation depending on the concentration of cyclodextrin. Determination of the concentration of 2-HP-β-CD in the duodenal aspirates clearly demonstrated that higher concentrations of 2-HP-β-CD were measured upon the intake of Sporanox® without water (Berben et al., 2018c). Based on these data, it was therefore assumed that the formation of higher order complexes between 2-HP-β-CD and itraconazole at higher 2-HP-β-CD concentrations resulted in stronger interactions between itraconazole and the excipient thereby reducing the free fraction of itraconazole available for absorption across the small intestinal epithelium. The intake of water reduced the affinity of itraconazole for the cyclodextrin inclusion complexes thereby resulting in the presence of the molecularly dissolved drug that is available for permeation. Indeed, the data obtained in the duodenum of the Diamod® demonstrated that higher apparent solubilities of itraconazole were obtained without water intake as compared to the condition with water intake. However, higher degrees of supersaturation, and hence molecularly dissolved drug, were present in the condition with water intake thereby explaining the comparable concentrations of permeated drug. As a conclusion, the Diamod® was capable to simulate *in vitro* the complex solubilization, supersaturation, precipitation, and permeation behavior of the Sporanox® solution as observed *in vivo*.

### 3.2 Effect of food on the gastrointestinal behavior of indinavir sulfate

**Fig. 3:**
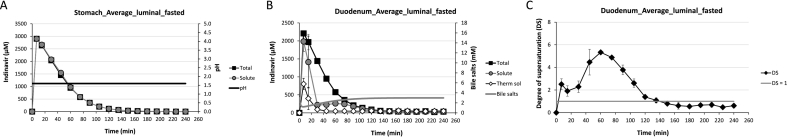
Average ± stdev (n = 3) concentration (μM) of total (black squares) and solute (grey circles) indinavir in the stomach (A) and duodenum (B) of the Diamod®, thermodynamic solubility (white diamonds) of indinavir in the duodenum (B) and degree of supersaturation (black diamonds) of indinavir in the duodenum (C) upon administration of indinavir sulfate under fasted state conditions. The pH value of the stomach (A) is represented by a black line whereas the total concentration of bile salts in the duodenum (B) is represented by a grey line.

In the fasted state, high total concentrations of indinavir were present in the stomach of the Diamod®. The total concentration of indinavir rapidly decreased in function of time due to the simulation of gastric secretion and rapid gastric monoexponential emptying. Comparison of the total and solute concentration indicated that indinavir was completely dissolved in the stomach compartment due to the low pH of 1.6 (Fig. 3A; Table 2). Complete dissolution of indinavir sulfate in the fasted state stomach has also been demonstrated in a small clinical study (Rubbens et al., 2016). Hence, high concentrations of dissolved indinavir were emptied into duodenum of the Diamod®. Determination of total and solute concentrations in the duodenum revealed that the rapid fasted state gastric emptying resulted in high peak concentrations of indinavir during the initial phase of the experiment. Furthermore, during the first 15 min indinavir was nearly completely dissolved in the duodenum resulting in rapid and substantial permeation of the compound into the acceptor compartment of the Diamod® (Fig. 3B and 5). The high solute concentrations of indinavir during the initial phases of gastric emptying were probably due to the short initial decrease in duodenal pH. Indeed, an initial decrease in duodenal pH from a value of around 6.7 to a value of approximately 3.0 was observed during the initial phase of gastric emptying in duodenal aspirates of fasted state individuals taking indinavir sulfate (Rubbens et al., 2016). After 30 min, the concentration of solute indinavir was lower than the total concentration in the duodenum of the Diamod® (Fig. 3B). This indicated that indinavir precipitated once the pH of the duodenum reverted back to 6.5 and this due to the pH shift from the acidic stomach to the more neutral duodenum. However, despite the very low solubility of indinavir in a neutral environment (50 μM at pH 6; Holmstock et al., 2013) and in FaHIF (84 ± 3 μM; Holmstock et al., 2013), solute concentrations above 200 μM were present in the Diamod® duodenum between 30 and 75 min of the experimental run. Determination of the thermodynamic solubility of indinavir in the duodenal samples and determination of the degree of supersaturation in those samples clearly demonstrated that indinavir supersaturated during transit through the duodenum of the Diamod® (Fig. 3B and C). Duodenal supersaturation of indinavir in the fasted state duodenum has been confirmed *in vivo* (Rubbens et al., 2016). The presence of supersaturated indinavir in the duodenum resulted in further permeation of the compound into the acceptor compartment of the Diamod® (Fig. 5). After 90 min, the concentrations of total and solute indinavir decreased in the duodenum due to duodenal emptying and dilution with duodenal secretions. The decreasing concentrations of solute indinavir in the duodenum resulted in lower rates of permeation finally resulting in a plateau phase of duodenal permeation (Fig. 5). Hence, the duodenal solute concentration and concentrations of permeated indinavir demonstrated that duodenal supersaturation and precipitation processes are kinetically interconnected with permeation processes in the Diamod®.

**Table 2:** Stomach, duodenal, and permeated AUC_0–240min_ of total and solute indinavir in the Diamod® upon administration of indinavir sulfate to the stomach under fasted state conditions, fed state conditions with ingestion of a high protein meal, fed state conditions with ingestion of a carbohydrate solution and high gall bladder activity, and fed state conditions with ingestion of a carbohydrate meal with low gall bladder activity. A comparison was made with the AUC _0-inf_ values measured in plasma samples of individuals under fasted or fed state conditions during an *in vivo* food effect study with indinavir sulfate (Carver et al., 1999).

	Diamod®					*In vivo*
	Stomach		Duodenum		Permeate	Plasma
	Average AUC_0–240min_ (μM*min) ± stdev		Average AUC_0–240min_ (μM*min) ± stdev		Average AUC_0–240min_ (μM*min) ± stdev	AUC_0-inf_ ratio (%) as compared to fasted
	Total	Solute	Total	Solute	Total	Total
Fasted	139,394.8 ± 2264.4	142,108.9 ± 1992.1	105,320.9 ± 10,083.5	55,044.5 ± 8425.6	1641.7 ± 245.9	100.00 ± 35.90
Fed_Ensure plus	194,925.5 ± 15,167.0	168,321.9 ± 7309.7	72,487.6 ± 1686.4	36,225.8 ± 1144.3	450.4 ± 65.9	31.83 ± 27.81
Fed_carbo_high bile	171,046.4 ± 8779.6	184,961.8 ± 10,113.8	70,237.6 ± 4085.4	64,432.4 ± 2626.7	1061.8 ± 40.4	Not tested
Fed_carbo_low bile	182,081.8 ± 797.1	178,266.3 ± 2007.0	74,067.6 ± 2724.1	59,651.5 ± 2963.8	1112.2 ± 25.8	55.00 ± 32.89

**Fig. 4:**
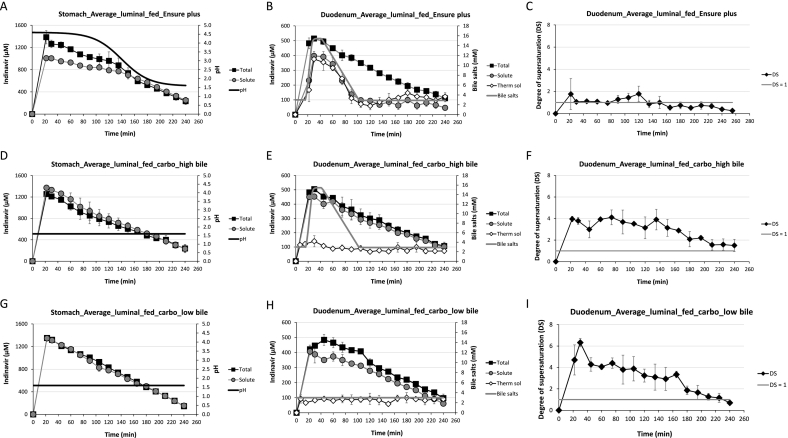
Average ± stdev (n = 3) concentration (μM) of total (black squares) and solute (grey circles) indinavir in the stomach (A, D, and G) and duodenum (B, E, and H) of the Diamod®, thermodynamic solubility (white diamonds) of indinavir in the duodenum (B, E, and H) and degree of supersaturation (black diamonds) of indinavir in the duodenum (C, F, and I) upon administration of indinavir sulfate under fed state conditions with a high protein meal (A, B, and C), fed state conditions with a carbohydrate meal and high bile salt secretions (D, E, and F) and fed state conditions with a carbohydrate meal and low bile salts concentrations (G, H, and I). The pH value of the stomach (A, D, and G) is represented by a black line whereas the total concentration of bile salts in the duodenum (B, E, and H) is represented by a grey line.

During the fed state Diamod® experiments simulating the ingestion of a high protein meal, indinavir sulfate was added to the stomach together with water (250 mL as in fasted state) and 400 mL of Ensure® Plus. Furthermore, to simulate the effect of a protein meal on the gastric pH, the pH of the Diamod® stomach was initially 4.6 after which a sigmoidal decrease to a value of 1.6 over 240 min was simulated through active pH control (Fig. 4A). The high volume of the fed state stomach resulted in the presence of lower maximum total concentrations of indinavir (C_max_ total = 1386.0 ± 120.11 μM) relative to the experiments under fasted state conditions (C_max_ total = 2902.4 ± 69.4 μM). The linear gastric emptying under fed conditions resulted in a slow removal of indinavir from the stomach as compared to fasted conditions during which the total concentration rapidly decreased in the stomach. The initial high pH of the stomach, under fed conditions, resulted in lower solute concentrations than total concentrations of indinavir, indicating that indinavir was not completely dissolved during the initial phase of stomach emptying (Fig. 4A; Table 2). As such, both solid and solute indinavir entered the duodenum during this phase of the experiment. After 150 min, indinavir was completely dissolved in the stomach due to the gradual decrease in pH. Hence, low concentrations of completely dissolved indinavir were transferred from the stomach to the duodenum in between 150 min and 240 min of the experimental run (Fig. 4A). Despite the incomplete dissolution of indinavir during the initial stomach phase and the high dilution of the compound due to the ingestion of Ensure® Plus, the slower gastric emptying kinetics under fed conditions resulted in a 1.2-fold increase in the overall AUC_0–240min_ of solute indinavir as compared to the fasted state experiments (Table 2).

**Fig. 5:**
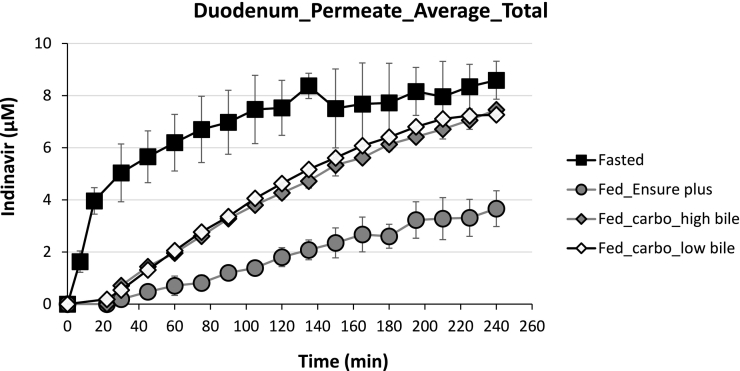
Average ± stdev (n = 3) concentration (μM) of permeated indinavir upon administration of indinavir sulfate under fasted state conditions (black squares) under fed state conditions with a high protein meal (grey circles), fed state conditions with a carbohydrate meal and high bile salt concentrations (grey diamonds) and fed state conditions with a carbohydrate meal and low bile salt concentrations (white diamonds).

In the duodenum, indinavir displayed a complex behavior. Due to the higher intragastric dilution, slower gastric emptying, and higher dilution with duodenal secretions lower maximum total concentrations and lower average total concentrations were present in the fed state (C_max_ total = 514.8 ± 16.5 µM; AUC_0–240min_ total = 72,487.6 ± 1686.4 μM*min) as compared to the fasted state (C_max_ total = 2210.0 ± 44.52 µM; AUC_0–240min_ total = 105,320.9 ± 10,083.5 μM*min) duodenum (Fig. 4B; Table 2). In the duodenum, the total concentration of indinavir was higher than the solute concentration indicating that indinavir precipitated upon entrance in the high pH environment of the fed duodenum (pH = 5.8). Interestingly, high concentrations of solute indinavir (C_max_ solute = 397.3 ± 29.8 μM) were present during the initial 95 min of the experiments even though indinavir was not completely dissolved in the stomach during this time frame (Fig. 4B). Theoretical calculations of the total bile salts concentrations in the duodenum demonstrated that the concentration of solute indinavir increased with increasing bile salt concentrations indicating that the compound is solubilized by micellar entrapment. Indeed, the high concentrations of bile salts at the start of the experiment resulted in solute concentrations in between 400 and 200 μM. Despite these very high solute concentrations, only minor permeation of indinavir in the acceptor compartment of the Diamod® was observed (Fig. 5). This clearly demonstrated that during this phase of the experiment indinavir was not present as a molecularly dissolved drug but as a solubilized colloid-associated drug preventing its absorption. Determination of the thermodynamic solubility and degree of supersaturation of indinavir in the duodenal samples taken from the Diamod® demonstrated that indinavir was completely solubilized in the duodenum and was not in a state of supersaturation (Fig. 4B and C), thereby explaining the low permeation of the compound during this time frame (Fig. 5). Once the concentration of bile salts decreased towards fasted state levels the difference between the total and solute concentration of indinavir increased. The lowered solubilizing capacity of the duodenal environment caused indinavir to precipitate. However, duodenal solute concentrations of around 100 μM were measured. The determination of the thermodynamic solubility and degree of supersaturation of indinavir in these samples taken from Diamod® indicated that indinavir was present at its thermodynamic solubility value and that no supersaturation occurred during these stage of the experimental run. (Fig. 4B and C). These data demonstrated the important interplay between the dynamics in fed state pH and fed state duodenum bile salts concentrations. Once the solubilizing effect of the bile salts was decreased, the pH of the stomach was lowered towards a value that allowed the complete dissolution of indinavir in the stomach, thereby delivering completely dissolved compound to the duodenum. In fact, solute concentrations after 105 min were lower than that measured during the period with high bile salt concentrations. In spite of this, a faster permeation of indinavir in the acceptor compartment of the Diamod® was observed indicating that indinavir was present in a state that was readily available for permeation. Overall, intake of indinavir sulfate under fed state conditions, simulating a high protein meal, resulted in a decrease in the average duodenal solute concentration AUC_0–240min_ of 34.2% as compared to fasted state conditions (Table 2). However, the dynamic interconnected duodenal dissolution and permeation in the Diamod® revealed that a high protein meal resulted in a decrease in the average permeated concentration AUC_0–240min_ of 72.6% (Table 2). Furthermore, under fed state conditions permeation only started to reach its plateau phase after 240 min whereas under fasted state conditions permeation mainly occurred during the first 90 min of the experiment. Similar food effects were observed during the clinical study of Carver et al. (1999).

During the fed state Diamod® simulating the ingestion of an isocaloric carbohydrate meal with high gall bladder activity (high bile salt concentrations), the pH in the stomach was continuously controlled at 1.6 (Fig. 4D). Carver et al. (1999) demonstrated that the ingestion of a carbohydrate meal had no effect on fed stomach pH as compared to the fasted state control conditions. As was the case for the fed experiments with a high protein meal the high volume of the stomach resulted in the presence of a lower maximum concentration of total indinavir (C_max_ total = 1225.5 ± 19.9 μM) as compared to the fasted stomach (Table 2). The low pH conditions present in the stomach resulted in the complete dissolution of indinavir resulting in the continuous gastric emptying of completely dissolved indinavir into the duodenum. Due to the low pH conditions and the slow linear emptying of dissolved indinavir out of the stomach, the average solute stomach concentration (AUC_0–240min_) was 1.2-fold higher as compared to the solute concentration in the fasted state stomach (Table 2).

The higher intragastric dilution, slower gastric emptying, and higher dilution by duodenal secretions resulted in lower total concentrations (C_max_ total = 504.3 ± 3.1 µM; AUC_0–240min_ total = 70,237 ± 4085.4 μM*min) as compared to the fasted state (C_max_ total = 2210.0 ± 44.52 µM; AUC_0–240min_ total = 105,320.9 ± 10,083.5 μM*min) in the duodenum (Table 2). The complete dissolution of indinavir sulfate in the stomach had beneficial effects on its behavior in the duodenum. Upon the pH shift from the stomach (pH 1.6) to the duodenum (pH 5.8) indinavir remained nearly completely dissolved as was demonstrated by the slightly lower concentrations of solute indinavir as compared to the total concentrations (Fig. 4E). Hence, the longer stomach dissolution and lower total concentration of indinavir in the duodenum resulted in less driving force for precipitation as compared to the fasted state experiments and the fed state experiments with elevated stomach pH. Indeed, determination of the thermodynamic solubility and degree of supersaturation of indinavir in the duodenal samples demonstrated that indinavir supersaturated throughout the transit through the duodenum (Fig. 4E and F). The absence of substantial precipitation resulted in high maximum solute concentrations (C_max_ = 452.55 ± 11.56 μM) and high average duodenal solute concentrations (AUC_0–240min_ = 64,432.4 ± 2626.7 μM*min) which were 1.17-fold higher than the average duodenal solute concentrations under fasted conditions (Table 2). Determination of the concentration of indinavir in the permeated fraction demonstrated that indinavir permeated throughout the experiment. Interestingly, the higher AUC_0–240 min_ of duodenal solute concentrations under the fed state condition with a carbohydrate meal did not result in higher permeation as compared to the fasted state condition. On the contrary, dosing of indinavir sulfate with the carbohydrate meal reduced the average concentration of permeated compound by 35.3%. Furthermore, the highest concentrations of permeated indinavir were obtained after 240 min under fed state conditions whereas permeation mainly occurred during the initial 90 min of the fasted state experiments (Fig. 5). These results demonstrated that the kinetics of dissolution and permeation in the Diamod® are interconnected as occurring *in vivo*. Since absorption of BCS class II compounds occurs through transcellular passive diffusion which is dictated by the concentration-gradient between the donor (duodenal lumen) and acceptor (blood stream) the initial very high duodenal concentrations of indinavir resulted in a very rapid and substantial permeation of the compound under fasted conditions. On the contrary, under fed conditions, the dynamics of gastric emptying and increased duodenal dilution resulted in lower solute concentrations thereby giving rise to a more steady linear permeation of the compound thereby reducing the overall exposure and increasing the Tmax. Indeed, the clinical study of Carver et al. (1999) demonstrated that the administration of indinavir sulfate together with a high carbohydrate meal resulted in a reduction in the average systemic exposure with 45% and increased the Tmax with approximately 3 h.

During the fed state Diamod® simulating the ingestion of an isocaloric carbohydrate meal with low gall bladder activity (low bile salts concentrations) comparable concentrations of total and solute indinavir were determined in the stomach and duodenum of the Diamod® (Fig. 4G and H). Furthermore, determination of the thermodynamic solubility and degree of supersaturation of indinavir in the duodenal samples of the Diamod® indicated that indinavir was supersaturating throughout the transit through the duodenum (Fig. 4H and I). Comparable thermodynamic solubility values and degree of supersaturation were obtained during this fed state run and the fed state run that simulated high bile salt concentrations. As such, it can be concluded that solubilization of indinavir by bile salts in the duodenum necessitates the presence of food compounds which are provided by Ensure® plus. This hypothesis was also confirmed by the fact that comparable concentrations of permeated indinavir were obtained during the fed run with high and low bile salt concentrations in the presence of a carbohydrate meal (Fig. 5). Hence, the formation of large colloidal inclusion complexes of indinavir, preventing the permeation of indinavir is depending on the presence of bile salts in combination with food compounds. Similar observations were obtained for fenofibrate that indicated that permeation of fenofibrate was not completely prevented by bile salts and that the presence of a nutritional drink (Fortimel) in combination with bile salts was necessary to form large colloidal inclusion complexes preventing the permeation of fenofibrate over a permeation barrier (Hens et al., 2015).

## 4 Conclusions

This study aimed at developing a dynamic gastrointestinal transfer model with physically interconnected permeation, namely the Diamod® and to assess the *in vivo*-predictive power of the Diamod® to study the gastrointestinal fate of different formulations and this under different prandial conditions. This study focused on *in vivo* cases for which the gastrointestinal behavior and interconnected permeation (solubility-permeability interplay) had a substantial effect on the final systemic exposure, thereby necessitating the use of an *in vitro* tool that simultaneously simulates gastrointestinal solubility and intestinal permeation in a stomach to duodenum transfer model. The Diamod® was optimized to accurately simulate the intraluminal total concentrations of a drug upon transfer through the gastrointestinal tract. Next to this, a physically interconnected permeation compartment was developed and implemented in this system with a permeation surface area-to-donor volume ratio of 2 cm^−1^. Most permeation setups described in literature have an area-to-volume ratio that is lower than 0.5 cm^−1^. These low ratios result in very low amounts of permeated drug in function of time thereby making it impossible to study permeation of a drug during physiologically relevant duodenal transit times using these systems. This was optimized in the Diamod® since the physiological area-to-volume ratio has been estimated between 1.9 cm^−1^ and 2.3 cm^−1^ (Berben et al., 2018a). By doing so, the Diamod® was capable to simulate the complex solubilization, supersaturation, precipitation, and permeation behavior of a Sporanox® solution taken with and without water as observed *in vivo*. Next to this, the Diamod® allowed to study the negative food effect for indinavir sulfate thereby resulting in a strong *in vitro-in vivo* correlation. Furthermore, the system highlighted both the role of increased gastric pH, slower gastric emptying, and micellar entrapment of indinavir sulfate under fed state conditions as compared to fasted state conditions on its final permeation thereby resulting into mechanistic insights into the *in vivo*-observed negative food effect.

## Declaration of Competing Interest

None.

## Data Availability

All generated data were included in the manuscript and are included in the text, tables, and figures
